# High-energy extracorporeal shock wave therapy for early stage femoral head osteonecrosis: A protocol of systematic review

**DOI:** 10.1097/MD.0000000000021300

**Published:** 2020-07-31

**Authors:** Qing-hui Ji, Shi-chen Liu, Jie Miao, Zhi-xin Ren, Yu-fei Yuan, Yan-bao Li

**Affiliations:** a First Ward of Orthopedis Department, First Affiliated Hospital of Jiamusi University, Jiamusi; b Fifth Ward of Orthopedis Department, Handan Central Hospital, Handan, China.

**Keywords:** extracorporeal shock wave, femoral head, high-energy, osteonecrosis

## Abstract

**Background:**

Published trials reported that high-energy extracorporeal shock wave therapy (HEEPSWT) can effectively treat early stage femoral head osteonecrosis (ESFHO). However, their results are still inconsistent. Thus, this study will systematically and comprehensively explore the effectiveness and safety of HEEPSWT for ESFHO.

**Methods:**

We will retrieve the electronic databases of Cochrane Library, EMBASE, PubMed, Web of Science, Cumulative Index to Nursing and Allied Health Literature, VIP database, and China National Knowledge Infrastructure from inception to the present. All randomized controlled trials that focusing on the effectiveness and safety of HEEPSWT for ESFHO will be considered. Two researchers will undertake literature selection, information collection, and risk of bias evaluation separately. If disagreements occur, we will invite a third researcher for consultation and a final decision will be made. Cochrane risk of bias tool, and Grades of Recommendation, Assessment, Development and Evaluation will be utilized to assess the risk of bias and quality of evidence, respectively. We will perform statistical analysis using RevMan 5.3 software.

**Results:**

This study will provide a detailed summary of exist evidence related to the effectiveness and safety of HEEPSWT for ESFHO.

**Conclusion:**

The results of this study synthesize the evidence regarding the HEEPSWT for ESFHO, which may help to guide clinical management in the future.

**Systematic review registration:**

INPLASY202060055.

## 1 Introduction

Femoral head osteonecrosis (FHO) is common disorder in young adults, which can affect any joint but mostly attack hip joint.^[1–4]^ It is characterized by decrease blood flow to the femoral head and thus results in cellular death, fractures, and collapse of joint surface.^[5,6]^ Despite it is well-known, its pathology remains poorly understood, and it is difficult to diagnose at early stage, also known as early stage FHO (ESFHO).^[7–9]^ Treatment approach of FHO depends on its stage, size, location, and its early diagnosis and preservation.^[8,9]^ However, the efficacy is still unsatisfied.

High-energy extracorporeal shock wave therapy (HEEPSWT) is an invasive treatment approach that has been utilized for the treatment of numerous orthopedic disorders.^[10–18]^ Several clinical trials have shown beneficial effects for patients with ESFHO.^[10,19–26]^ However, there is not systematic review focusing on effectiveness and safety of HEEPSWT for ESFHO. Thus, the objective of this systematic review is to appraise the effectiveness and safety of HEEPSWT for patients with ESFHO.

## 2 Methods and analysis

### 2.1 Study registration

This protocol was registered on INPLASY202060055. It has been conducted according to the guideline of Preferred Reporting Items for Systematic Reviews and Meta-Analysis Protocol statement guidelines.^[27,28]^

### 2.2 Eligibility criteria for study selection

#### 2.2.1 Types of study

We will include all potential randomized controlled trials (RCTs) focusing on the effectiveness and safety of HEEPSWT for ESFHO in spite of language and publication status. We will exclude any other studies, such as animal studies, case report, case series, reviews, comments, non-clinical trials, non-controlled trials, and non-RCTs.

#### 2.2.2 Types of participant

This study will fully consider patients who were clinically diagnosed as ESFHO for inclusion inconsiderate their country, race, sex, and age.

#### 2.2.3 Types of intervention

In the experimental group, all subjects underwent single HEEPSWT intervention as their solely management.

In the control group, all patients received any treatments, but not any forms of HEEPSWT, will be included.

#### 2.2.4 Types of outcome measurements

##### 2.2.4.1 Primary outcome

Pain intensity of hip or knee joints (as assessed by Numerical Rating Scale or any other pain scales).

##### 2.2.4.2 Secondary outcomes

Functional status and limitation of hip or knee joints (as evaluated by Western Ontario and McMaster Universities Osteoarthritis Index or other related indexes);

Health-related quality of life (as identified by 12-Item Short-Form Health Survey or other connected tools); and

Adverse events.

### 2.3 Literature search

We will identify the following electronic databases from conception to the present: Cochrane Library, EMBASE, PUBMED, Web of Science, Cumulative Index to Nursing and Allied Health Literature, VIP database, and China National Knowledge Infrastructure. No limitation will be applied to language and publication status. We will consider any potential RCTs that investigated the effectiveness and safety of HEEPSWT for ESFHO. The sample of search strategy for Cochrane Library is crated (Table 1). Similar search strategy with details will also be built for other electronic databases.

**Table 1:**
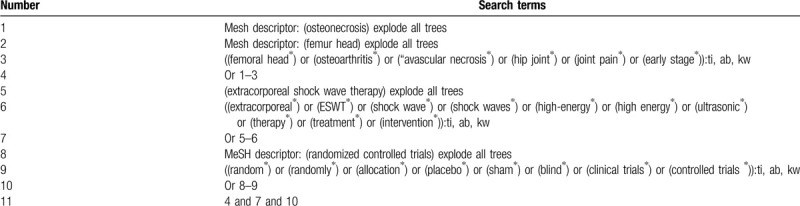
Search strategy for Cochrane Library.

In addition, we will search dissertations, conference abstracts, and reference lists of any relevant reviews to avoid missing any potential literature.

### 2.4 Study selection

Two researchers will independently select all records using predefined eligibility criteria. It will be conducted by a pilot test to check inter-rater reliability and will correct each selecting step. At the first step, the tiles/abstracts of all searched citations will be scanned, and all unconnected records will be removed. At the second step, the remaining potential studies will be cautiously read through the full manuscripts against all inclusion criteria when necessary. Any divergences will be solved by discussion with the help of a third researcher. The whole procedure of study selection will be presented in a flow diagram.

### 2.5 Data extraction and management

Two independent researchers will extract data from each eligible trial using a predefined standardized data extraction form. The extracted data comprises of reference identification, author information, patient characteristics, study design, sample size, study methods, details of interventions and comparators, endpoints at different time points, results, findings, adverse events, conflict of interests, and funding information. Any disagreements will be figured out via discussion with the help of a third researcher.

### 2.6 Missing data dealing with

When there is insufficient or missing data, all corresponding or related authors of primary trials will be contacted to obtain it. If such data is not available, we will analyze the outcome data using an intention-to-treat analysis. We will also explore its potential affects in the discussion section.

### 2.7 Study quality assessment

Study quality of each included trial will be estimated based on the guidelines of Cochrane Risk of Bias Tool by 2 independent researchers. It comprises of 7 specific fields, and each one is further rated as low risk of bias, unclear risk of bias, and high risk of bias. Any differences in assessment will be resolved through consultation or discussion with the help of another researcher.

### 2.8 Statistical analysis

This study will utilize RevMan 5.3 software (Cochrane Community, London, UK) to perform statistical analysis.

#### 2.8.1 Treatment effect measurement

Results regarding the pain intensity, functional status, limitation of knee or hip joints, and health-related quality of life, the outcome data will be expressed as mean difference, or standardized mean difference and 95% confidence intervals (CIs). Regarding the incidence of adverse events, it will be calculated as risk ratio and 95% CIs.

#### 2.8.2 Heterogeneity assessment

The extent of statistical heterogeneity is investigated with *I*^2^ test. If *I*^2^ ≤ 50%, we will estimate it as having minor or low heterogeneity. If *I*^2^ > 50%, we will estimate it as having significant heterogeneity.

#### 2.8.3 Data synthesis

If sufficient data will be collected with minor heterogeneity across the trials, we will undertake a meta-analysis according to the similar conditions of study and patient characteristics, specifics of interventions and controls, and outcome measurements. If we find significant heterogeneity across the studies, we will perform a subgroup analysis. If the meta-analysis is deemed not to be conducted, we will present outcome results as a narrative summary.

#### 2.8.4 Subgroup analysis

We will perform subgroup analysis to identify any possible sources of substantial heterogeneity based on the variations in characteristics, different treatments, controls, outcome measurements.

#### 2.8.5 Sensitivity analysis

We will conduct sensitivity analysis to examine the robustness of the merged outcomes by removing trials with low quality.

#### 2.8.6 Publication bias

We will plan to run funnel plot and Egger test to detect if there are any reporting biases when we include at least 10 eligible trials.

#### 2.8.7 Summary of evidence

The quality of evidence for main outcome will be assessed by the Grading of Recommendations Assessment, Development, and Evaluation System approach.^[29,30]^ We will present its results in the “summary of findings” tables. If any conflicts occur, we will invite another researcher to solve them via discussion.

#### 2.8.8 Dissemination and ethics

This study will not use individual patient data, thus no ethical approval is required. We will publish this study through a peer-reviewed journal.

## 3 Discussion

ESFHO is a frequency disorder in young adults.^[1–4]^ Although it is a well-known condition, it is still not early to elaborate its pathology and diagnosis at early stage.^[7–9]^ Previous clinical trials suggested HEEPSWT can effectively treat patients with ESFHO. However, their results are contradictory, and no systematic review explores this topic.

To the best of our knowledge, the results of this systematic review will fill a crucial knowledge gap of HEEPSWT for ESFHO. We hope the findings of this study will benefit both patients and clinicians. Additionally, this study may also help guide future research and relevant head-to head RCTs.

## Author contributions

**Conceptualization:** Qing-hui Ji, Shi-chen Liu, Yu-fei Yuan, Yan-bao Li.

**Data curation:** Qing-hui Ji, Zhi-xin Ren, Yan-bao Li.

**Formal analysis:** Qing-hui Ji, Shi-chen Liu, Jie Miao, Yan-bao Li.

**Investigation:** Yan-bao Li.

**Methodology:** Qing-hui Ji, Jie Miao, Zhi-xin Ren, Yu-fei Yuan.

**Project administration:** Yan-bao Li.

**Resources:** Qing-hui Ji, Shi-chen Liu, Jie Miao, Zhi-xin Ren, Yu-fei Yuan.

**Software:** Qing-hui Ji, Shi-chen Liu, Jie Miao, Zhi-xin Ren, Yu-fei Yuan.

**Supervision:** Yan-bao Li.

**Validation:** Qing-hui Ji, Shi-chen Liu, Jie Miao, Yan-bao Li.

**Visualization:** Qing-hui Ji, Shi-chen Liu, Jie Miao, Zhi-xin Ren, Yu-fei Yuan, Yan-bao Li.

**Writing – original draft:** Qing-hui Ji, Shi-chen Liu, Zhi-xin Ren, Yu-fei Yuan, Yan-bao Li.

**Writing – review & editing:** Qing-hui Ji, Shi-chen Liu, Jie Miao, Zhi-xin Ren, Yu-fei Yuan, Yan-bao Li.
